# Daratumumab plus lenalidomide and dexamethasone in transplant-ineligible newly diagnosed multiple myeloma: frailty subgroup analysis of MAIA

**DOI:** 10.1038/s41375-021-01488-8

**Published:** 2022-01-02

**Authors:** Thierry Facon, Gordon Cook, Saad Z. Usmani, Cyrille Hulin, Shaji Kumar, Torben Plesner, Cyrille Touzeau, Nizar J. Bahlis, Supratik Basu, Hareth Nahi, Hartmut Goldschmidt, Hang Quach, Mohamad Mohty, Christopher P. Venner, Katja Weisel, Noopur Raje, Benjamin Hebraud, Karim Belhadj-Merzoug, Lotfi Benboubker, Olivier Decaux, Salomon Manier, Denis Caillot, Jon Ukropec, Huiling Pei, Rian Van Rampelbergh, Clarissa M. Uhlar, Rachel Kobos, Sonja Zweegman

**Affiliations:** 1 University of Lille, CHU Lille, Service des Maladies du Sang, Lille, France; 2 Leeds Cancer Centre, Leeds Teaching Hospitals Trust, Leeds, UK; 3 Levine Cancer Institute/Atrium Health, Charlotte, NC USA; 4 Department of Hematology, Hôpital Haut Lévêque, University Hospital, Pessac, France; 5 Department of Hematology, Mayo Clinic Rochester, Rochester, MN USA; 6 Vejle Hospital and University of Southern Denmark, Vejle, Denmark; 7 Centre Hospitalier Universitaire, Nantes, France; 8 University of Calgary, Arnie Charbonneau Cancer Research Institute, Calgary, AB Canada; 9 The Royal Wolverhampton Hospitals NHS Trust, University of Wolverhampton, Wolverhampton, UK; 10 Karolinska Institute, Department of Medicine, Division of Hematology, Karolinska University Hospital at Huddinge, Stockholm, Sweden; 11 University Clinic Heidelberg, International Medicine V and National Center of Tumor Diseases (NCT), Heidelberg, Germany; 12 University of Melbourne, St. Vincent’s Hospital, Melbourne, VIC Australia; 13 Sorbonne University, Department of Hematology, Saint-Antoine Hospital, Paris, France; 14 Cross Cancer Institute, University of Alberta, Edmonton, AB Canada; 15 Department of Oncology, Hematology and Bone Marrow Transplantation With Section of Pneumology, University Medical Center Hamburg-Eppendorf, Hamburg, Germany; 16 Department of Hematology/Oncology, Massachusetts General Hospital, Boston, MA USA; 17 Institut Universitaire du Cancer and University Hospital, Toulouse, France; 18 Hémopathies Lymphoïdes, Hôpital Henri Mondor, Créteil, France; 19 CHRU de Tours, Hôpital de Bretonneau, Tours, France; 20 Clinical Haematology Department, University of Rennes, CHU Rennes, CIC INSERM 1414, Rennes, France; 21 CHU Dijon, Hôpital du Bocage, Dijon, France; 22 Janssen Global Medical Affairs, Horsham, PA USA; 23 Janssen Research & Development, LLC, Titusville, NJ USA; 24 Janssen Research & Development, Beerse, Belgium; 25 Janssen Research & Development, LLC, Spring House, PA USA; 26 Janssen Research & Development, LLC, Raritan, NJ USA; 27 Department of Hematology, Amsterdam UMC, Vrije Universiteit Amsterdam, Cancer Center Amsterdam, Amsterdam, The Netherlands

**Keywords:** Myeloma, Molecularly targeted therapy

## Abstract

In the phase 3 MAIA study of patients with transplant-ineligible newly diagnosed multiple myeloma (NDMM), daratumumab plus lenalidomide/dexamethasone (D-Rd) improved progression-free survival (PFS) versus lenalidomide/dexamethasone (Rd). We present a subgroup analysis of MAIA by frailty status. Frailty assessment was performed retrospectively using age, Charlson comorbidity index, and baseline Eastern Cooperative Oncology Group performance status score. Patients were classified as fit, intermediate, non-frail (fit + intermediate), or frail. Of the randomized patients (D-Rd, *n* = 368; Rd, *n* = 369), 396 patients were non-frail (D-Rd, 196 [53.3%]; Rd, 200 [54.2%]) and 341 patients were frail (172 [46.7%]; 169 [45.8%]). After a 36.4-month median follow-up, non-frail patients had longer PFS than frail patients, but the PFS benefit of D-Rd versus Rd was maintained across subgroups: non-frail (median, not reached [NR] vs 41.7 months; hazard ratio [HR], 0.48; *P* < 0.0001) and frail (NR vs 30.4 months; HR, 0.62; *P* = 0.003). Improved rates of complete response or better and minimal residual disease (10^–5^) negativity were observed for D-Rd across subgroups. The most common grade 3/4 treatment-emergent adverse event in non-frail and frail patients was neutropenia (non-frail, 45.4% [D-Rd] and 37.2% [Rd]; frail, 57.7% and 33.1%). These findings support the clinical benefit of D-Rd in transplant-ineligible NDMM patients enrolled in MAIA, regardless of frailty status.

## Introduction

Daratumumab, a human IgGκ monoclonal antibody targeting CD38 with a direct on-tumor [1–4] and immunomodulatory [5–7] mechanism of action, is approved in many countries as monotherapy for relapsed or refractory multiple myeloma (RRMM) patients and in combination with standard-of-care regimens for RRMM and newly diagnosed multiple myeloma (NDMM) patients [8–17]. In the primary analysis of the phase 3 MAIA study in transplant-ineligible NDMM patients (28.0-month median follow-up), daratumumab plus lenalidomide/dexamethasone (D-Rd) versus lenalidomide/dexamethasone (Rd) significantly improved progression-free survival (PFS) and induced deeper responses, including improved rates of complete response or better (≥CR) and minimal residual disease (MRD) negativity [14]. With longer treatment duration (36.4-month median follow-up), D-Rd continued to demonstrate a PFS benefit and deeper responses [18]. The median age was 73 years, and 43.6% of patients were aged ≥75 years [18]. In both the primary and updated analyses, D-Rd improved PFS, even in patients aged ≥75 years [14, 18].

Although D-Rd improved outcomes in older patients, such patients often vary widely in fitness level [19, 20]. The ability or inability to tolerate cancer treatment regimens logically impacts clinical outcomes and is dependent on overall health as determined by the functional status of numerous organ systems [20, 21]. Therefore, analyses of a treatment based on frailty status should be more informative than analyses based solely on age. A frailty scoring system developed by the International Myeloma Working Group (IMWG) classifies patients into 3 frailty subgroups—fit, intermediate, and frail—based on age, comorbidities (Charlson comorbidity index [CCI]), patient-evaluated self-care (Katz Activities of Daily Living [ADL] scale), and household management (Lawton Instrumental Activities of Daily Living [IADL] scale) assessments [20]. However, use of the scoring system was not feasible with the MAIA study, as the MAIA study did not assess patients using ADL and IADL scales.

Similarly, the FIRST trial did not assess patients using ADL and IADL scales, which led to the development of a frailty scale based on age, CCI (using medical history of patients), and the physician-evaluated Eastern Cooperative Oncology Group performance status (ECOG PS) score in a retrospective subgroup analysis of the trial [19]. The frailty scale, similar to the IMWG scale, allows classification of patients into fit, intermediate, and frail subgroups; the 3-subgroup frailty classification was also used in a frailty subgroup analysis of the A.R.R.O.W. study [22]. Use of the frailty scale was further simplified to classify patients into only 2 subgroups—frail and non-frail [19]. Both 3-subgroup and simplified 2-subgroup frailty classifications were shown to be predictive of clinical outcomes in transplant-ineligible NDMM patients [19, 20, 23].

We present a subgroup analysis of MAIA comparing D-Rd versus Rd across frailty subgroups based on the 3-subgroup and simplified 2-subgroup frailty classifications [19].

## Patients and methods

### Study design and patients

MAIA (ClinicalTrials.gov Identifier: NCT02252172) is a randomized, open-label, phase 3 trial. The study was conducted in accordance with the principles of the Declaration of Helsinki and the International Conference on Harmonisation Good Clinical Practice guidelines. Independent ethics committees or institutional review boards at each institution approved the study protocol. All patients provided written informed consent.

The complete methodology of MAIA has been previously described [14]. Briefly, patients with documented NDMM ineligible for high-dose chemotherapy with autologous stem cell transplant due to age ≥65 years or comorbidities, an ECOG PS score ≤2, and a creatinine clearance (CrCl) ≥30 mL/min were eligible.

### Treatment

Patients (*N* = 737) were randomized 1:1 to D-Rd or Rd; randomization was stratified by International Staging System (ISS) disease stage (I vs II vs III), geographic region (North America vs other), and age (<75 vs ≥75 years). During each 28-day cycle, all patients received lenalidomide 25 mg (10 mg recommended if CrCl 30–50 mL/min) orally on Days 1–21 and dexamethasone 40 mg (20 mg if aged >75 years or body mass index <18.5 kg/m^2^) orally on Days 1, 8, 15, and 22. Patients in the D-Rd cohort received daratumumab 16 mg/kg intravenously once weekly during Cycles 1–2, every 2 weeks during Cycles 3–6, and then every 4 weeks thereafter. Treatment in both cohorts continued until disease progression or unacceptable toxicity.

### Frailty evaluation

Frailty assessment was performed retrospectively on all patients using age, CCI (based on retrospective review of each patient’s medical history), and baseline ECOG PS score (Supplementary Table 1) [19]. Frailty scores were used to classify patients into fit (0), intermediate (1), or frail (≥2) subgroups. Frailty status was further simplified into 2 categories: total–non-frail (0–1; a combination of the fit and intermediate subgroups) and frail (≥2). Patients within the total–non-frail and frail subgroups were further divided by ISS stage (I/II vs III). Patients with missing data were excluded from frailty evaluation.

### Assessments and statistical analyses

The primary endpoint was PFS. Post-hoc analyses were performed by patient frailty status. Efficacy endpoints were assessed based on the intent-to-treat population (all randomized patients). Safety was assessed in the safety population (patients who received ≥1 dose of study treatment). See Supplementary Information for details on statistical analyses.

## Results

### Patient disposition and treatment

A total of 737 patients were randomized to D-Rd (*n* = 368) or Rd (*n* = 369). Frailty scores were retrospectively calculated for all randomized patients; 146 (19.8%) patients were classified as fit (D-Rd, 68 [18.5%]; Rd, 78 [21.1%]), 250 (33.9%) patients were intermediate (128 [34.8%]; 122 [33.1%]), and 341 (46.3%) patients were frail (172 [46.7%]; 169 [45.8%]). The total–non-frail subgroup (a combination of the fit and intermediate subgroups) included 396 (53.7%) patients (D-Rd, 196 [53.3%]; Rd, 200 [54.2%]). In the fit subgroup, 1 patient randomized to Rd did not receive treatment, whereas all patients in the intermediate subgroup received treatment. In the frail subgroup, 4 patients randomized to D-Rd and 3 patients randomized to Rd did not receive treatment. Demographics and baseline characteristics were generally balanced between the treatment cohorts within each frailty subgroup (Table 1).

**Table 1:** Demographics and baseline characteristics^a^.

	Non-frail^b^						Frail	
	Fit (19.8%^c^; *n* = 146/737)		Intermediate (33.9%^c^; *n* = 250/737)		Total–non-frail^b^ (53.7%^c^; *n* = 396/737)		Frail (46.3%^c^; *n* = 341/737)	
	D-Rd (18.5%^d^; *n* = 68/368)	Rd (21.1%^e^; *n* = 78/369)	D-Rd (34.8%^d^; *n* = 128/368)	Rd (33.1%^e^; *n* = 122/369)	D-Rd (53.3%^d^; *n* = 196/368)	Rd (54.2%^e^; *n* = 200/369)	D-Rd (46.7%^d^; *n* = 172/368)	Rd (45.8%^e^; *n* = 169/369)
Age, years, *n* (%)								
Median (range)	70.0 (65–75)	71.0 (64–75)	72.0 (50–80)	72.0 (61–80)	71.0 (50–80)	72.0 (61–80)	77.0 (57–90)	77.0 (45–89)
<65	0	2 (2.6)	2 (1.6)	1 (0.8)	2 (1.0)	3 (1.5)	2 (1.2)	1 (0.6)
65–<70	27 (39.7)	26 (33.3)	29 (22.7)	27 (22.1)	56 (28.6)	53 (26.5)	18 (10.5)	20 (11.8)
70–<75	36 (52.9)	44 (56.4)	62 (48.4)	62 (50.8)	98 (50.0)	106 (53.0)	32 (18.6)	25 (14.8)
≥75	5 (7.4)	6 (7.7)	35 (27.3)	32 (26.2)	40 (20.4)	38 (19.0)	120 (69.8)	123 (72.8)
≥80	0	0	6 (4.7)	4 (3.3)	6 (3.1)	4 (2.0)	60 (34.9)	67 (39.6)
Sex, *n* (%)								
Female	37 (54.4)	31 (39.7)	63 (49.2)	64 (52.5)	100 (51.0)	95 (47.5)	79 (45.9)	79 (46.7)
ECOG PS score, *n* (%)								
0	68 (100.0)	78 (100.0)	39 (30.5)	27 (22.1)	107 (54.6)	105 (52.5)	20 (11.6)	18 (10.7)
1	0	0	89 (69.5)	95 (77.9)	89 (45.4)	95 (47.5)	89 (51.7)	92 (54.4)
≥2	0	0	0	0	0	0	63 (36.6)	59 (34.9)
ISS stage, *n* (%)^f^								
I	27 (39.7)	34 (43.6)	37 (28.9)	34 (27.9)	64 (32.7)	68 (34.0)	34 (19.8)	35 (20.7)
II	27 (39.7)	31 (39.7)	62 (48.4)	58 (47.5)	89 (45.4)	89 (44.5)	74 (43.0)	67 (39.6)
III	14 (20.6)	13 (16.7)	29 (22.7)	30 (24.6)	43 (21.9)	43 (21.5)	64 (37.2)	67 (39.6)
Type of measurable disease, *n* (%)								
IgG	39 (57.4)	52 (66.7)	83 (64.8)	80 (65.6)	122 (62.2)	132 (66.0)	103 (59.9)	99 (58.6)
IgA	14 (20.6)	13 (16.7)	19 (14.8)	19 (15.6)	33 (16.8)	32 (16.0)	32 (18.6)	34 (20.1)
Other^g^	2 (2.9)	3 (3.8)	2 (1.6)	2 (1.6)	4 (2.0)	5 (2.5)	5 (2.9)	5 (3.0)
Detected in urine only	8 (11.8)	5 (6.4)	13 (10.2)	14 (11.5)	21 (10.7)	19 (9.5)	19 (11.0)	15 (8.9)
Detected as serum free light chain only	5 (7.4)	5 (6.4)	11 (8.6)	7 (5.7)	16 (8.2)	12 (6.0)	13 (7.6)	16 (9.5)
CrCl (mL/min), *n* (%)								
≥90	12 (17.6)	18 (23.1)	25 (19.5)	22 (18.0)	37 (18.9)	40 (20.0)	24 (14.0)	20 (11.8)
60–<90	37 (54.4)	42 (53.8)	59 (46.1)	65 (53.3)	96 (49.0)	107 (53.5)	49 (28.5)	60 (35.5)
30–<60	19 (27.9)	18 (23.1)	44 (34.4)	34 (27.9)	63 (32.1)	52 (26.0)	92 (53.5)	86 (50.9)
<30	0	0	0	1 (0.8)	0	1 (0.5)	7 (4.1)	3 (1.8)
Cytogenetic profile^h^								
*N*	57	71	109	105	166	176	153	147
Standard risk, *n* (%)	48 (84.2)	62 (87.3)	95 (87.2)	93 (88.6)	143 (86.1)	155 (88.1)	128 (83.7)	124 (84.4)
High risk, *n* (%)^i^	9 (15.8)	9 (12.7)	14 (12.8)	12 (11.4)	23 (13.9)	21 (11.9)	25 (16.3)	23 (15.6)
del17p	3 (5.3)	3 (4.2)	9 (8.3)	10 (9.5)	12 (7.2)	13 (7.4)	13 (8.5)	16 (10.9)
t(4;14)	4 (7.0)	6 (8.5)	5 (4.6)	2 (1.9)	9 (5.4)	8 (4.5)	12 (7.8)	4 (2.7)
t(14;16)	2 (3.5)	0	1 (0.9)	1 (1.0)	3 (1.8)	1 (0.6)	1 (0.7)	4 (2.7)
Median time since initial diagnosis of MM (range), months	1.05 (0.2–8.7)	0.94 (0.2–14.5)	1.03 (0.1–8.7)	0.80 (0.2–4.3)	1.03 (0.1–8.7)	0.89 (0.2–14.5)	0.90 (0.2–13.3)	0.95 (0.0–9.2)

*D-Rd* daratumumab plus lenalidomide/dexamethasone, *Rd* lenalidomide/dexamethasone, *ECOG PS* Eastern Cooperative Oncology Group performance status, *ISS* International Staging System, *CrCl* creatinine clearance, *MM* multiple myeloma, *ITT* intent-to-treat.

^a^Percentages in the table were calculated using the number of patients in each treatment cohort per frailty subgroup of the ITT population (fit: D-Rd, *n* = 68; Rd, *n* = 78; intermediate: D-Rd, *n* = 128; Rd, *n* = 122; total–non-frail: D-Rd, *n* = 196; Rd, *n* = 200; frail: D-Rd, *n* = 172; Rd, *n* = 169) as the denominator, unless otherwise indicated.

^b^Non-frail subgroup consists of fit and intermediate patients.

^c^Percentage was calculated using the number of patients in the ITT population as the denominator.

^d^Percentage was calculated using the number of patients in the D-Rd cohort of the ITT population as the denominator.

^e^Percentage was calculated using the number of patients in the Rd cohort of the ITT population as the denominator.

^f^Based on the combination of serum β_2_-microglobulin and albumin.

^g^Includes IgD, IgE, IgM, and biclonal.

^h^Cytogenetic risk was based on fluorescence in situ hybridization or karyotype analysis. Percentages were calculated using the number of patients in each treatment cohort per frailty subgroup with available baseline cytogenetic data as the denominator.

^i^Patients with high-risk cytogenetics had a del17p, t(14;16), or t(4;14) abnormality.

The dispositions of patients according to frailty status are summarized in Table 2. For both the D-Rd and Rd cohorts, the proportion of patients who discontinued treatment was highest in the frail subgroup; the proportion was lower in the D-Rd cohort versus the Rd cohort across frailty subgroups. Among patients randomized to D-Rd, a higher proportion of patients discontinued treatment during the first 12 months of treatment in the frail subgroup versus other frailty subgroups (Table 2). Overall, the 2 most common reasons for treatment discontinuation with D-Rd and Rd in all frailty subgroups were progressive disease and adverse event (AE). Median (range) time to treatment discontinuation with D-Rd and Rd was 22.3 (2.5–38.2) and 17.3 (0.3–35.3) months, respectively, in the fit subgroup, 16.4 (0.7–41.4) and 10.4 (0.5–35.0) months in the intermediate subgroup, 19.1 (0.7–41.4) and 12.0 (0.3–35.3) months in the total–non-frail subgroup, and 13.4 (0.1–39.4) and 12.1 (0.03–41.9) months in the frail subgroup. Permanent lenalidomide discontinuations occurred less frequently in the total–non-frail versus frail subgroup with D-Rd and Rd and occurred more frequently with D-Rd versus Rd across frailty subgroups, with AE as the most common reason (Table 3). Median (range) time to permanent lenalidomide discontinuation was longer in the total–non-frail versus frail subgroup with D-Rd and Rd and with D-Rd versus Rd across frailty subgroups (fit, 21.9 [4.0–38.0] vs 11.4 [2.3–34.8] months, respectively; intermediate, 19.5 [1.3–38.1] vs 11.0 [1.4–33.8] months; total–non-frail, 21.3 [1.3–38.1] vs 11.0 [1.4–34.8] months; frail, 14.4 [0.2–37.8] vs 7.6 [0.03–41.9] months).

**Table 2:** Patient disposition (ITT population)^a^.

	Non-frail^b^						Frail	
	Fit (19.8%^c^; *n* = 146/737)		Intermediate (33.9%^c^; *n* = 250/737)		Total–non-frail^b^ (53.7%^c^; *n* = 396/737)		Frail (46.3%^c^; *n* = 341/737)	
	D-Rd (18.5%^d^; *n* = 68/368)	Rd (21.1%^e^; *n* = 78/369)	D-Rd (34.8%^d^; *n* = 128/368)	Rd (33.1%^e^; *n* = 122/369)	D-Rd (53.3%^d^; *n* = 196/368)	Rd (54.2%^e^; *n* = 200/369)	D-Rd (46.7%^d^; *n* = 172/368)	Rd (45.8%^e^; *n* = 169/369)
Patients who discontinued treatment, *n* (%)	20 (29.4)	45 (57.7)	45 (35.2)	74 (60.7)	65 (33.2)	119 (59.5)	78 (45.3)	114 (67.5)
Reason for discontinuation, *n* (%)								
PD	14 (20.6)	21 (26.9)	25 (19.5)	35 (28.7)	39 (19.9)	56 (28.0)	32 (18.6)	43 (25.4)
AE	5 (7.4)	12 (15.4)	9 (7.0)	21 (17.2)	14 (7.1)	33 (16.5)	17 (9.9)	32 (18.9)
Non-compliance with study drug^f^	1 (1.5)	4 (5.1)	5 (3.9)	7 (5.7)	6 (3.1)	11 (5.5)	8 (4.7)	12 (7.1)
Death	0	2 (2.6)	5 (3.9)	3 (2.5)	5 (2.6)	5 (2.5)	18 (10.5)	15 (8.9)
Physician decision	0	5 (6.4)	0	7 (5.7)	0	12 (6.0)	2 (1.2)	6 (3.6)
Patient withdrawal	0	1 (1.3)	0	1 (0.8)	0	2 (1.0)	0	4 (2.4)
Lost to follow-up	0	0	0	0	0	0	0	2 (1.2)
Other	0	0	1 (0.8)	0	1 (0.5)	0	1 (0.6)	0
Patients who discontinued treatment during the first 12 months, *n* (%)	5 (7.4)	19 (24.4)	16 (12.5)	42 (34.4)	21 (10.7)	61 (30.5)	38 (22.1)	56 (33.1)
Reason for discontinuation during the first 12 months, *n* (%)								
PD	3 (4.4)	6 (7.7)	8 (6.3)	16 (13.1)	11 (5.6)	22 (11.0)	12 (7.0)	12 (7.1)
AE	1 (1.5)	6 (7.7)	5 (3.9)	15 (12.3)	6 (3.1)	21 (10.5)	10 (5.8)	20 (11.8)
Non-compliance with study drug^f^	1 (1.5)	3 (3.8)	2 (1.6)	3 (2.5)	3 (1.5)	6 (3.0)	4 (2.3)	8 (4.7)
Death	0	1 (1.3)	1 (0.8)	3 (2.5)	1 (0.5)	4 (2.0)	10 (5.8)	9 (5.3)
Physician decision	0	3 (3.8)	0	4 (3.3)	0	7 (3.5)	2 (1.2)	3 (1.8)
Patient withdrawal	0	0	0	1 (0.8)	0	1 (0.5)	0	3 (1.8)
Lost to follow-up	0	0	0	0	0	0	0	1 (0.6)

*ITT* intent-to-treat, *D-Rd* daratumumab plus lenalidomide/dexamethasone, *Rd* lenalidomide/dexamethasone, *PD* progressive disease, *AE* adverse event.

^a^Percentages in the table were calculated using the number of patients in each treatment cohort per frailty subgroup of the ITT population (fit: D-Rd, *n* = 68; Rd, *n* = 78; intermediate: D-Rd, *n* = 128; Rd, *n* = 122; total–non-frail: D-Rd, *n* = 196; Rd, *n* = 200; frail: D-Rd, *n* = 172; Rd, *n* = 169) as the denominator, unless otherwise indicated.

^b^Non-frail subgroup consists of fit and intermediate patients.

^c^Percentage was calculated using the number of patients in the ITT population as the denominator.

^d^Percentage was calculated using the number of patients in the D-Rd cohort of the ITT population as the denominator.

^e^Percentage was calculated using the number of patients in the Rd cohort of the ITT population as the denominator.

^f^Based on reason “Patient refused further study treatment.”

**Table 3:** Lenalidomide discontinuations and dose modifications (safety population)^a^.

	Non-frail^b^						Frail	
	Fit (19.9%^c^; *n* = 145/729)		Intermediate (34.3%^c^; *n* = 250/729)		Total–non-frail^b^ (54.2%^c^; *n* = 395/729)		Frail (45.8%^c^; *n* = 334/729)	
	D-Rd (18.7%^d^; *n* = 68/364)	Rd (21.1%^e^; *n* = 77/365)	D-Rd (35.2%^d^; *n* = 128/364)	Rd (33.4%^e^; *n* = 122/365)	D-Rd (53.8%^d^; *n* = 196/364)	Rd (54.5%^e^; *n* = 199/365)	D-Rd (46.2%^d^; *n* = 168/364)	Rd (45.5%^e^; *n* = 166/365)
Lenalidomide permanent discontinuation, *n* (%)	13 (19.1)	8 (10.4)	24 (18.8)	17 (13.9)	37 (18.9)	25 (12.6)	45 (26.8)	23 (13.9)
Reason for discontinuation, *n* (%)								
AE	12 (17.6)	5 (6.5)	18 (14.1)	6 (4.9)	30 (15.3)	11 (5.5)	37 (22.0)	14 (8.4)
Other	1 (1.5)	3 (3.9)	6 (4.7)	11 (9.0)	7 (3.6)	14 (7.0)	8 (4.8)	9 (5.4)
Lenalidomide dose delay, *n* (%)	13 (19.1)	7 (9.1)	15 (11.7)	11 (9.0)	28 (14.3)	18 (9.0)	31 (18.5)	13 (7.8)
Reason for delay, *n* (%)								
AE	7 (10.3)	3 (3.9)	8 (6.3)	5 (4.1)	15 (7.7)	8 (4.0)	14 (8.3)	8 (4.8)
Other	7 (10.3)	4 (5.2)	7 (5.5)	8 (6.6)	14 (7.1)	12 (6.0)	21 (12.5)	7 (4.2)
Lenalidomide dose skipped, *n* (%)	50 (73.5)	47 (61.0)	101 (78.9)	78 (63.9)	151 (77.0)	125 (62.8)	140 (83.3)	109 (65.7)
Reason for skipping, *n* (%)								
AE	39 (57.4)	32 (41.6)	85 (66.4)	66 (54.1)	124 (63.3)	98 (49.2)	117 (69.6)	77 (46.4)
Other	30 (44.1)	31 (40.3)	54 (42.2)	41 (33.6)	84 (42.9)	72 (36.2)	81 (48.2)	66 (39.8)
Lenalidomide dose reduced, *n* (%)	53 (77.9)	40 (51.9)	83 (64.8)	70 (57.4)	136 (69.4)	110 (55.3)	118 (70.2)	88 (53.0)
Reason for reduction, *n* (%)								
AE	51 (75.0)	38 (49.4)	74 (57.8)	63 (51.6)	125 (63.8)	101 (50.8)	108 (64.3)	73 (44.0)
Other	3 (4.4)	4 (5.2)	13 (10.2)	11 (9.0)	16 (8.2)	15 (7.5)	22 (13.1)	24 (14.5)

*D-Rd* daratumumab plus lenalidomide/dexamethasone, *Rd* lenalidomide/dexamethasone, *AE* adverse event.

^a^Percentages in the table were calculated using the number of patients in each treatment cohort per frailty subgroup of the safety population (fit: D-Rd, *n* = 68; Rd, *n* = 77; intermediate: D-Rd, *n* = 128; Rd, *n* = 122; total–non-frail: D-Rd, *n* = 196; Rd, *n* = 199; frail: D-Rd, *n* = 168; Rd, *n* = 166) as the denominator, unless otherwise indicated.

^b^Non-frail subgroup consists of fit and intermediate patients.

^c^Percentage was calculated using the number of patients in the safety population as the denominator.

^d^Percentage was calculated using the number of patients in the D-Rd cohort of the safety population as the denominator.

^e^Percentage was calculated using the number of patients in the Rd cohort of the safety population as the denominator.

Median (range) duration of treatment was longer in the total–non-frail versus frail subgroup with D-Rd and Rd and was longer with D-Rd versus Rd across frailty subgroups (fit, 34.6 [2.5–47.2] vs 29.6 [0.3–49.0] months, respectively; intermediate, 33.2 [0.7–47.8] vs 20.1 [0.5–47.9] months; total–non-frail, 33.6 [0.7–47.8] vs 25.8 [0.3–49.0] months; frail, 31.1 [0.1–49.0] vs 20.7 [0.03–41.9] months). The median relative dose intensity (RDI) of daratumumab was similar across frailty subgroups (≥98.0%; Table 4). The median RDI of lenalidomide and dexamethasone was lower with D-Rd versus Rd in all frailty subgroups. Among patients who permanently discontinued lenalidomide, the median RDI of lenalidomide with D-Rd and Rd was 60.0% and 88.4%, respectively, in the fit subgroup, 71.4% and 75.0% in the intermediate subgroup, 63.8% and 80.0% in the total–non-frail subgroup, and 68.8% and 69.5% in the frail subgroup. A reduced starting dose of lenalidomide (<25 mg), in most cases due to a CrCl of ≤50 mL/min per study protocol, was given less frequently to total–non-frail versus frail patients with D-Rd and Rd (fit, 12 [17.6%] and 9 [11.5%], respectively; intermediate, 24 [18.8%] and 17 [13.9%]; total–non-frail, 36 [18.4%] and 26 [13.0%]; frail, 76 [44.2%] and 58 [34.3%]). These patients started lenalidomide dosing at 10 mg, except for 1 frail patient (Rd cohort) who received 15 mg and 1 frail patient (D-Rd cohort) who received 5 mg. Incidences of lenalidomide dose modifications were higher for D-Rd versus Rd across frailty subgroups (Table 3). The median cumulative dose of lenalidomide was 525 mg in each of the first 6 cycles with D-Rd and Rd for all frailty subgroups except for the D-Rd cohort in the frail subgroup (Cycle 1, 338 mg; Cycle 2, 315 mg; Cycle 3, 300 mg; Cycle 4, 308 mg; Cycles 5 and 6, 210 mg) and the Rd cohort of the frail subgroup (Cycle 2, 513 mg).

**Table 4:** Summary of RDI (safety population)^a^.

	Non-frail^b^						Frail	
	Fit (19.9%^c^; *n* = 145/729)		Intermediate (34.3%^c^; *n* = 250/729)		Total–non-frail^b^ (54.2%^c^; *n* = 395/729)		Frail (45.8%^c^; *n* = 334/729)	
	D-Rd (18.7%^d^; *n* = 68/364)	Rd (21.1%^e^; *n* = 77/365)	D-Rd (35.2%^d^; *n* = 128/364)	Rd (33.4%^e^; *n* = 122/365)	D-Rd (53.8%^d^; *n* = 196/364)	Rd (54.5%^e^; *n* = 199/365)	D-Rd (46.2%^d^; *n* = 168/364)	Rd (45.5%^e^; *n* = 166/365)
Lenalidomide RDI, %								
* N*	66	75	125	120	191	195	148	153
Mean (SD)	74.7 (31.8)	79.7 (20.7)	75.9 (29.7)	80.9 (28.6)	75.5 (30.4)	80.4 (25.8)	66.4 (27.8)	85.1 (34.0)
Median (range)	70.4 (20.9–235.7)	84.7 (24.9–100.0)	79.7 (7.9–241.2)	86.6 (20.6–238.6)	77.2 (7.9–241.2)	86.4 (20.6–238.6)	65.4 (9.5–175.0)	92.9 (4.8–238.1)
Dexamethasone RDI, %								
Mean (SD)	73.3 (21.9)	77.6 (21.5)	74.3 (22.4)	81.0 (19.3)	74.0 (22.2)	79.7 (20.2)	80.2 (20.0)	83.3 (21.2)
Median (range)	71.5 (30.8–100.0)	83.3 (29.9–100.8)	77.5 (22.9–100.0)	85.8 (27.2–100.0)	75.0 (22.9–100.0)	84.8 (27.2–100.8)	85.8 (28.0–110.7)	90.3 (18.9–154.5)
Daratumumab RDI, %								
Mean (SD)	97.8 (5.7)	–	96.6 (7.7)	–	97.0 (7.1)	–	94.5 (13.1)	–
Median (range)	98.4 (61.5–104.7)	–	98.2 (38.5–107.0)	–	98.2 (38.5–107.0)	–	98.0 (3.2–107.0)	–

*RDI* relative dose intensity, *D*-*Rd* daratumumab plus lenalidomide/dexamethasone, *Rd* lenalidomide/dexamethasone, *SD* standard deviation.

^a^Percentages in the table were calculated using the number of patients in each treatment cohort per frailty subgroup of the safety population (fit: D-Rd, *n* = 68; Rd, *n* = 77; intermediate: D-Rd, *n* = 128;Rd, *n* = 122; total–non-frail: D-Rd, *n* = 196; Rd, *n* = 199; frail: D-Rd, *n* = 168; Rd, *n* = 166) as the denominator, unless otherwise indicated.

^b^Non-frail subgroup consists of fit and intermediate patients.

^c^Percentage was calculated using the number of patients in the safety population as the denominator.

^d^Percentage was calculated using the number of patients in the D-Rd cohort of the safety population as the denominator.

^e^Percentage was calculated using the number of patients in the Rd cohort of the safety population as the denominator.

### Efficacy

**Fig. 1:**
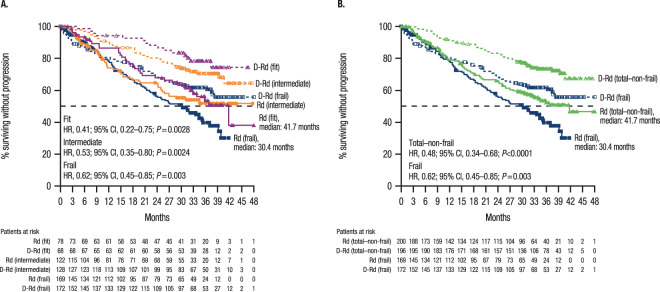
PFS. PFS in the (**A**) fit, intermediate, and frail subgroups and (**B**) total–non-frail and frail subgroups. PFS progression-free survival, D-Rd daratumumab plus lenalidomide/dexamethasone, Rd lenalidomide/dexamethasone, HR hazard ratio, CI confidence interval.

**Fig. 2:**
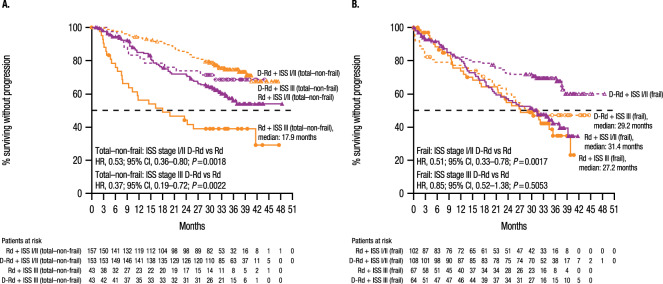
PFS subdivided by ISS stage. PFS subdivided by ISS stage in the (**A**) total–non-frail subgroup and (**B**) frail subgroup. PFS progression-free survival, ISS International Staging System, D-Rd daratumumab plus lenalidomide/dexamethasone, Rd lenalidomide/dexamethasone, HR hazard ratio, CI confidence interval.

After a 36.4-month median follow-up, the PFS benefit of D-Rd versus Rd was maintained in all frailty subgroups: fit (median, not reached [NR] vs 41.7 months; hazard ratio [HR], 0.41; 95% confidence interval [CI], 0.22–0.75; *P* = 0.0028), intermediate (NR in both cohorts; HR, 0.53; 95% CI, 0.35–0.80; *P* = 0.0024), total–non-frail (NR vs 41.7 months; HR, 0.48; 95% CI, 0.34–0.68; *P* < 0.0001), and frail (NR vs 30.4 months; HR, 0.62; 95% CI, 0.45–0.85; *P* = 0.003; Fig. 1A and B). The 36-month PFS rate was higher in the D-Rd cohort in all subgroups, with decreasing rates from fit to frail (fit, D-Rd, 78.3% vs Rd, 53.6%; intermediate, 70.4% vs 51.7%; total–non-frail, 73.2% vs 52.1%; frail, 61.5% vs 39.5%). In the total–non-frail and frail subgroups subdivided by ISS stage (I/II vs III), the PFS benefit of D-Rd versus Rd was also maintained in most subgroups, with the exception of frail + ISS III (Fig. 2A and B). Regardless of lenalidomide starting dose, the PFS benefit of D-Rd versus Rd was observed in the intent-to-treat population (25 mg: median, NR vs 35.4 months; HR, 0.51; 95% CI, 0.38–0.68; *P* < 0.0001; <25 mg: NR vs 26.8 months; HR, 0.56; 95% CI, 0.37–0.85; *P* = 0.0053) and the total–non-frail subgroup (25 mg: NR in both cohorts; HR, 0.51; 95% CI, 0.35–0.76; *P* = 0.0006; <25 mg: NR vs 19.8 months; HR, 0.35; 95% CI, 0.16–0.72; *P* = 0.0048); PFS benefit was less pronounced in frail patients who received a lenalidomide starting dose of <25 mg (25 mg: NR vs 31.4 months; HR, 0.51; 95% CI, 0.33–0.80; *P* = 0.0026; <25 mg: NR vs 26.9 months; HR, 0.72; 95% CI, 0.44–1.18; *P* = 0.1891; Supplementary Fig. 1).

Five (7.4%) versus 12 (15.4%) fit patients and 21 (16.4%) versus 34 (27.9%) intermediate patients had died in the D-Rd and Rd cohorts, respectively; patient deaths were less frequent in the total–non-frail subgroup (26 [13.3%] vs 46 [23.0%]) compared with the frail subgroup (59 [34.3%] vs 57 [33.7%]).

Higher overall response rates (ORRs) were achieved with D-Rd versus Rd across frailty subgroups, with the total–non-frail subgroup achieving higher ORRs compared with the frail subgroup in each treatment cohort (fit, 100.0% vs 83.3%; *P* = 0.0004; intermediate, 96.9% vs 85.2%; *P* = 0.0012; total–non-frail, 98.0% vs 84.5%; *P* < 0.0001; frail, 87.2% vs 78.1%; *P* = 0.0265). Higher ≥CR rates and MRD-negativity (10^–5^ sensitivity threshold) rates were achieved with D-Rd versus Rd across frailty subgroups; total–non-frail patients had higher MRD-negativity rates than frail patients in the D-Rd cohort (33.2% vs 23.8%, respectively), and in the Rd cohort, total–non-frail and frail patients had similarly low MRD-negativity rates (8.5% vs 10.1%; Table 5). Median time to ≥CR was shorter with D-Rd versus Rd (fit, 10.3 vs 12.1 months; intermediate, 11.3 vs 13.8 months; total–non-frail, 10.4 vs 13.3 months; frail, 10.6 vs 11.5 months).

**Table 5:** Response and MRD-negativity rates (ITT population)^a^.

	Non-frail^b^									Frail		
	Fit (19.8%^c^; *n* = 146/737)			Intermediate (33.9%^c^; *n* = 250/737)			Total–non-frail^b^ (53.7%^c^; *n* = 396/737)			Frail (46.3%^c^; *n* = 341/737)		
	D-Rd (18.5%^d^; *n* = 68/368)	Rd (21.1%^e^; *n* = 78/369)	*P* value	D-Rd (34.8%^d^; *n* = 128/368)	Rd (33.1%^e^; *n* = 122/369)	*P* value	D-Rd (53.3%^d^; *n* = 196/368)	Rd (54.2%^e^; *n* = 200/369)	*P* value	D-Rd (46.7%^d^; *n* = 172/368)	Rd (45.8%^e^; *n* = 169/369)	*P* value
ORR	68 (100.0)	65 (83.3)	0.0004	124 (96.9)	104 (85.2)	0.0012	192 (98.0)	169 (84.5)	<0.0001	150 (87.2)	132 (78.1)	0.0265
≥CR, *n* (%)	38 (55.9)	17 (21.8)	<0.0001	69 (53.9)	31 (25.4)	<0.0001	107 (54.6)	48 (24.0)	<0.0001	75 (43.6)	52 (30.8)	0.0144
sCR	27 (39.7)	13 (16.7)	0.0019	44 (34.4)	9 (7.4)	<0.0001	71 (36.2)	22 (11.0)	<0.0001	49 (28.5)	29 (17.2)	0.0129
CR	11 (16.2)	4 (5.1)		25 (19.5)	22 (18.0)		36 (18.4)	26 (13.0)		26 (15.1)	23 (13.6)	
≥VGPR, *n* (%)	59 (86.8)	42 (53.8)	<0.0001	108 (84.4)	70 (57.4)	<0.0001	167 (85.2)	112 (56.0)	<0.0001	128 (74.4)	91 (53.8)	<0.0001
VGPR	21 (30.9)	25 (32.1)		39 (30.5)	39 (32.0)		60 (30.6)	64 (32.0)		53 (30.8)	39 (23.1)	
PR	9 (13.2)	23 (29.5)		16 (12.5)	34 (27.9)		25 (12.8)	57 (28.5)		22 (12.8)	41 (24.3)	
SD	0	11 (14.1)		3 (2.3)	18 (14.8)		3 (1.5)	29 (14.5)		8 (4.7)	26 (15.4)	
PD	0	0		0	0		0	0		1 (0.6)	0	
NE	0	2 (2.6)		1 (0.8)	0		1 (0.5)	2 (1.0)		13 (7.6)	11 (6.5)	
MRD negative (10^–5^), n (%)	23 (33.8)	6 (7.7)	<0.0001	42 (32.8)	11 (9.0)	<0.0001	65 (33.2)	17 (8.5)	<0.0001	41 (23.8)	17 (10.1)	0.0008

*MRD* minimal residual disease, *ITT* intent-to-treat, *D-Rd* daratumumab plus lenalidomide/dexamethasone, *Rd* lenalidomide/dexamethasone, *ORR* overall response rate, *≥CR* complete response or better, *sCR* stringent complete response, *CR* complete response, *≥VGPR* very good partial response or better, *VGPR* very good partial response, *PR* partial response, *SD* stable disease, *PD* progressive disease, *NE* not evaluable.

^a^Percentages in the table were calculated using the number of patients in each treatment cohort per frailty subgroup of the ITT population (fit: D-Rd, *n* = 68; Rd, *n* = 78; intermediate: D-Rd, *n* = 128; Rd, *n* = 122; total–non-frail: D-Rd, *n* = 196; Rd, *n* = 200; frail: D-Rd, *n* = 172; Rd, *n* = 169) as the denominator, unless otherwise indicated.

^b^Non-frail subgroup consists of fit and intermediate patients.

^c^Percentage was calculated using the number of patients in the ITT population as the denominator.

^d^Percentage was calculated using the number of patients in the D-Rd cohort of the ITT population as the denominator.

^e^Percentage was calculated using the number of patients in the Rd cohort of the ITT population as the denominator.

### Safety

Most common (≥10% patients) grade 3/4 treatment-emergent AEs (TEAEs) are summarized in Table 6 (see Supplementary Table 2 for all grade 3/4 TEAEs reported in >1 patient in either treatment cohort within each frailty subgroup). The grade 3/4 TEAE incidence was higher in the frail subgroup versus all other frailty subgroups (fit, D-Rd, 85.3% and Rd, 79.2%; intermediate, 91.4% and 85.2%; total–non-frail, 89.3% and 82.9%; frail, 94.6% and 89.2%). The incidence of grade 3/4 non-hematologic TEAEs was also higher in the frail subgroup versus all other frailty subgroups (fit, D-Rd, 70.6% and Rd, 75.3%; intermediate, 78.9% and 73.8%; total–non-frail, 76.0% and 74.4%; frail, 83.9% and 81.9%). The most common grade 3/4 TEAE with D-Rd and Rd in all frailty subgroups was neutropenia (fit, 44.1% and 28.6%, respectively; intermediate, 46.1% and 42.6%; total–non-frail, 45.4% and 37.2%; frail, 57.7% and 33.1%). Use of growth factors was most common in the frail subgroup and was more common with D-Rd versus Rd across frailty subgroups (Supplementary Table 3). Among the 7.6% of intermediate patients with a CCI ≥1 (D-Rd, *n* = 13; Rd, *n* = 6), grade 3/4 TEAEs were reported in 12 (92.3%) D-Rd and 6 (100.0%) Rd patients. Among the 32.6% of frail patients with a CCI ≥1 (D-Rd, *n* = 56; Rd, *n* = 53), grade 3/4 TEAEs were reported in 46 (82.1%) D-Rd and 46 (86.8%) Rd patients.

**Table 6:** Most common grade 3/4 (≥10% of patients) TEAEs and TEAEs with an outcome of death (>1 patient; safety population)^a^.

	Non-frail^b^						Frail	
	Fit (19.9%^c^; *n* = 145/729)		Intermediate (34.3%^c^; *n* = 250/729)		Total–non-frail^b^ (54.2%^c^; *n* = 395/729)		Frail (45.8%^c^; *n* = 334/729)	
	D-Rd (18.7%^d^; *n* = 68/364)	Rd (21.1%^e^; *n* = 77/365)	D-Rd (35.2%^d^; *n* = 128/364)	Rd (33.4%^e^; *n* = 122/365)	D-Rd (53.8%^d^; *n* = 196/364)	Rd (54.5%^e^; *n* = 199/365)	D-Rd (46.2%^d^; *n* = 168/364)	Rd (45.5%^e^; *n* = 166/365)
Total number of patients with grade 3/4 TEAE, *n* (%)	58 (85.3)	61 (79.2)	117 (91.4)	104 (85.2)	175 (89.3)	165 (82.9)	159 (94.6)	148 (89.2)
Hematologic, *n* (%)								
Neutropenia	30 (44.1)	22 (28.6)	59 (46.1)	52 (42.6)	89 (45.4)	74 (37.2)	97 (57.7)	55 (33.1)
Lymphopenia	7 (10.3)	7 (9.1)	18 (14.1)	14 (11.5)	25 (12.8)	21 (10.6)	31 (18.5)	18 (10.8)
Leukopenia	7 (10.3)	2 (2.6)	11 (8.6)	10 (8.2)	18 (9.2)	12 (6.0)	22 (13.1)	9 (5.4)
Anemia	4 (5.9)	11 (14.3)	17 (13.3)	24 (19.7)	21 (10.7)	35 (17.6)	28 (16.7)	40 (24.1)
Thrombocytopenia	4 (5.9)	3 (3.9)	8 (6.3)	12 (9.8)	12 (6.1)	15 (7.5)	17 (10.1)	18 (10.8)
Non-hematologic, *n* (%)								
Infections	16 (23.5)	22 (28.6)	46 (35.9)	30 (24.6)	62 (31.6)	52 (26.1)	70 (41.7)	46 (27.7)
Pneumonia	7 (10.3)	5 (6.5)	13 (10.2)	11 (9.0)	20 (10.2)	16 (8.0)	33 (19.6)	17 (10.2)
Cataract	10 (14.7)	8 (10.4)	11 (8.6)	9 (7.4)	21 (10.7)	17 (8.5)	13 (7.7)	19 (11.4)
Pulmonary embolism	8 (11.8)	5 (6.5)	6 (4.7)	9 (7.4)	14 (7.1)	14 (7.0)	7 (4.2)	5 (3.0)
Hypokalemia	7 (10.3)	5 (6.5)	12 (9.4)	10 (8.2)	19 (9.7)	15 (7.5)	18 (10.7)	20 (12.0)
Hyperglycemia	2 (2.9)	2 (2.6)	13 (10.2)	4 (3.3)	15 (7.7)	6 (3.0)	12 (7.1)	8 (4.8)
Total number of patients with TEAE with outcome of death, *n* (%)	1 (1.5)	3 (3.9)	6 (4.7)	4 (3.3)	7 (3.6)	7 (3.5)	20 (11.9)	20 (12.0)
General physical health deterioration	0	0	0	1 (0.8)	0	1 (0.5)	2 (1.2)	1 (0.6)
Pneumonia	0	0	0	0	0	0	2 (1.2)	3 (1.8)
Myocardial infarction	0	0	0	2 (1.6)	0	2 (1.0)	1 (0.6)	1 (0.6)
Cardiac arrest	0	0	0	0	0	0	1 (0.6)	2 (1.2)
Sepsis	0	0	0	0	0	0	0	3 (1.8)

*TEAE* treatment emergent adverse event, *D-Rd* daratumumab plus lenalidomide/dexamethasone, *Rd* lenalidomide/dexamethasone.

^a^Percentages in the table were calculated using the number of patients in each treatment cohort per frailty subgroup of the safety population (fit: D-Rd, *n* = 68; Rd, *n* = 77; intermediate: D-Rd, *n* = 128; Rd, *n* = 122; total–non-frail: D-Rd, *n* = 196; Rd, *n* = 199; frail: D-Rd, *n* = 168; Rd, *n* = 166) as the denominator, unless otherwise indicated.

^b^Non-frail subgroup consists of fit and intermediate patients.

^c^Percentage was calculated using the number of patients in the safety population as the denominator.

^d^Percentage was calculated using the number of patients in the D-Rd cohort of the safety population as the denominator.

^e^Percentage was calculated using the number of patients in the Rd cohort of the safety population as the denominator.

In both treatment cohorts, the serious TEAE incidence was higher in the frail subgroup versus all other frailty subgroups (fit, D-Rd, 34 [50.0%] and Rd, 47 [61.0%]; intermediate, 89 [69.5%] and 79 [64.8%]; total–non-frail, 123 [62.8%] and 126 [63.3%]; frail, 125 [74.4%] and 121 [72.9%]). Similarly, the incidence of serious non-hematologic TEAEs was higher in the frail subgroup versus all other frailty subgroups (fit, D-Rd, 50.0% and Rd, 61.0%; intermediate, 69.5% and 63.1%; total–non-frail, 62.8% and 62.3%; frail, 73.8% and 72.3%). The most common serious TEAE with D-Rd and Rd was pneumonia (fit, 8 [11.8%] and 6 [7.8%], respectively; intermediate, 13 [10.2%] and 12 [9.8%]; total–non-frail, 21 [10.7%] and 18 [9.0%]; frail, 30 [17.9%] and 14 [8.4%]). Among intermediate patients with a CCI ≥1, serious TEAEs were reported in 10 (76.9%) D-Rd and 3 (50.0%) Rd patients. Among frail patients with a CCI ≥1, serious TEAEs were reported in 41 (73.2%) D-Rd and 36 (67.9%) Rd patients.

In both treatment cohorts, treatment discontinuations due to any grade TEAEs in the safety population were higher in the frail subgroup versus all other frailty subgroups (fit, D-Rd, 5 [7.4%] and 11 [14.3%]; intermediate, 8 [6.3%] and 20 [16.4%]; total–non-frail, 13 [6.6%] and 31 [15.6%]; frail, 17 [10.1%] and 32 [19.3%]). The TEAE that led to treatment discontinuation most frequently was fatigue (2 patients each in the fit and intermediate subgroups; Rd, 0 patients) in the D-Rd cohort and pulmonary embolism (2 patients in the fit subgroup and 1 patient in the intermediate subgroup; D-Rd, 0 patients) in the Rd cohort. Infections leading to discontinuations were rare; pneumonia was a reason for discontinuation in only the frail subgroup (1 patient per treatment cohort).

The frail subgroup had an increased incidence of deaths and TEAEs resulting in death versus all other frailty subgroups. Deaths were reported in 11.7% of fit patients (D-Rd, 5 [7.4%]; Rd, 12 [15.6%]), 22.0% of intermediate patients (21 [16.4%]; 34 [27.9%]), 18.2% of total–non-frail patients (26 [13.3%]; 46 [23.1%]), and 34.1% of frail patients (57 [33.9%]; 57 [34.3%]). TEAEs resulting in death occurred in 1 (1.5%) fit patient in the D-Rd cohort and 3 (3.9%) fit patients in the Rd cohort, 6 (4.7%) and 4 (3.3%) intermediate patients, 7 (3.6%) and 7 (3.5%) total–non-frail patients, and 20 (11.9%) and 20 (12.0%) frail patients (Table 6). Disease progression as the primary cause of death was reported in 5.5% of fit patients (D-Rd, 3 [4.4%]; Rd, 5 [6.5%]), 10.8% of intermediate patients (10 [7.8%]; 17 [13.9%]), 8.9% of total–non-frail patients (13 [6.6%]; 22 [11.1%]), and 12.3% of frail patients (23 [13.7%]; 18 [10.8%]). Deaths occurring within 60 days of receipt of the first dose of study treatment were reported in 0.4% of intermediate patients (Rd, 1 [0.8%]) and in 4.8% of frail patients (D-Rd, 10 [6.0%]; Rd, 6 [3.6%]). Deaths occurring within 90 days of receipt of the first dose of study treatment were reported in 0.8% of intermediate patients (Rd, 2 [1.6%]) and 6.0% of frail patients (D-Rd, 13 [7.7%]; Rd, 7 [4.2%]); AEs were the primary cause of death in 1 of these 2 intermediate patients and in most of these frail patients (D-Rd, 12 [92.3%]; Rd, 6 [85.7%]).

## Discussion

After >3 years of follow-up, D-Rd demonstrated improved efficacy versus Rd in transplant-ineligible NDMM patients, regardless of frailty status. Compared with the total–non-frail subgroup, patients in the frail subgroup had poorer outcomes in both treatment cohorts. Nevertheless, D-Rd reduced the risk of disease progression or death by 52% in total–non-frail patients and by 38% in frail patients. The PFS results demonstrated that D-Rd leads to outcomes in frail patients that are at least as good as those observed with Rd in fit patients. Median PFS was NR in the D-Rd cohort for any frailty subgroup, whereas the Rd cohort did reach this milestone in both the total–non-frail (41.7 months) and frail (30.4 months) subgroups. Importantly, a greater PFS benefit of D-Rd over Rd was seen in total–non-frail and frail patients with lower ISS disease stage (I/II) and total–non-frail patients in the ISS stage III category. A greater PFS benefit of D-Rd over Rd was also seen in total–non-frail and frail patients who received a lenalidomide starting dose of <25 mg, with the benefit less pronounced in frail patients. Regardless of frailty status, deep responses were achieved with D-Rd versus Rd, with improved rates of ≥CR and MRD negativity. Consistent with the findings in the FIRST trial [19], the use of the ECOG PS score–containing frailty scale predicted clinical outcomes in transplant-ineligible NDMM patients, with frail patients demonstrating worse prognosis in terms of PFS and response rates versus total–non-frail patients.

The safety profile of D-Rd in frailty subgroups was generally consistent with the overall population of MAIA [14]; although higher rates of grade 3/4 neutropenia and pneumonia were observed with D-Rd in the frail subgroup than in the total–non-frail subgroup, these events were clinically manageable. The frail subgroup had an increased incidence of hematologic and non-hematologic grade 3/4 TEAEs, serious TEAEs, and deaths in both treatment cohorts versus the total–non-frail subgroup, but this was not unexpected based on the additional comorbidities frequently associated with frailty. Among patients in the frail subgroup, a higher incidence of grade 3/4 neutropenia was observed with D-Rd versus Rd. Across frailty subgroups, the incidences of treatment discontinuation overall and due to AEs were higher with Rd versus D-Rd, while the incidences of lenalidomide dose modifications overall and due to AEs were higher with D-Rd versus Rd. These findings may indicate that clinicians were more likely to modify the dose of lenalidomide due to AEs, such as neutropenia, with D-Rd versus Rd, as patients in the D-Rd cohort were also receiving daratumumab. Although there was no clear association observed between a CCI ≥1 and higher rates of grade 3/4 TEAEs or serious TEAEs in frail patients, a greater proportion of deaths occurred within 60 and 90 days of receipt of the first dose of study treatment in the frail subgroup versus other frailty subgroups, and almost all of these deaths were due to AEs; overall, the TEAE with an outcome of death observed most frequently in the frail subgroup was pneumonia (D-Rd, 1.2%; Rd, 1.8%). Consistent with the increased incidence of grade 3/4 TEAEs in the frail subgroup versus other frailty subgroups in both treatment cohorts, the frail subgroup had a shorter duration of treatment and a higher frequency of treatment discontinuations. The median RDI of lenalidomide was lower with D-Rd versus Rd in all frailty subgroups; this difference was most pronounced in the frail subgroup. The median RDI of daratumumab was nearly identical across frailty subgroups. A reduced starting dose of lenalidomide (<25 mg) was given more frequently to daratumumab-treated patients in all frailty subgroups, with the highest frequency reported in the frail subgroup. Growth factors were used most commonly in the frail subgroup and were more commonly used with D-Rd versus Rd across frailty subgroups.

In a separate analysis of MAIA age subgroups, D-Rd reduced the risk of disease progression or death by 37% in patients aged ≥75 years and by 50% in patients aged <75 years, similar to results reported in the frail and total–non-frail subgroups [24]. Thus, the results of the current MAIA frailty subgroup analysis combined with the MAIA age subgroup analysis highlight the key role D-Rd can play as first-line treatment in transplant-ineligible NDMM patients. In the real-world treatment of NDMM, each additional line of therapy is associated with worse outcomes [25]. In transplant-ineligible NDMM patients, attrition was found to be as high as 50% per line of therapy, with the high attrition level associated with older age and poor comorbidity status [26]. These data suggest that the most effective treatment regimen should be provided upfront, as frail patients may not have the opportunity to be treated with additional lines of therapy later.

A frailty subgroup analysis using the same frailty scale as our study was conducted on the phase 3 ALCYONE study of daratumumab plus bortezomib/melphalan/prednisone (D-VMP) versus bortezomib/melphalan/prednisone (VMP) [27]. After a 40.1-month median follow-up, the overall survival (OS) and PFS benefit of D-VMP versus VMP was observed in all frailty subgroups. With OS data not yet mature at the time of this analysis, the effect of frailty on OS remains to be seen in MAIA. Patients in ALCYONE in the D-VMP cohort received single-agent daratumumab starting in Cycle 10; thus, a better safety profile in frail daratumumab-treated patients was observed in ALCYONE compared with in MAIA. The results of these frailty subgroup analyses of MAIA and ALCYONE support the use of daratumumab-based regimens in transplant-ineligible NDMM patients.

This study provides validation of the simplified frailty score implemented in the FIRST trial [19]. The retrospective assessment of frailty score was a limitation of this study. Retrospective CCI calculations were based on reported medical history, which may contain missing data and result in underestimating or overestimating the number of patients in each frailty subgroup. Additionally, the ECOG PS score parameter used for frailty score calculations in our study is more subjective, with susceptibility to intra- and inter-observer bias, compared with the ADL and IADL scales used in the IMWG scoring system [28, 29]. Furthermore, while the frailty scale used in our study is based on parameters that are routinely assessed in clinical practice and is therefore practical for clinical use, the use of comprehensive frailty assessments that more accurately reflect biological or functional frailty will remain important for the further optimization of treatment strategies for frail patients [29]. Finally, patients with an ECOG PS score ≥3 and patients with comorbidities that may interfere with the study procedures were excluded from MAIA; the inclusion and exclusion criteria for the study limits the generalizability of these results to more frail patients seen in clinical practice.

In conclusion, improved efficacy with D-Rd versus Rd was observed across frailty subgroups, consistent with the overall study population. Our findings, although based on a retrospective assessment of frailty, support the clinical benefit of D-Rd in patients with transplant-ineligible NDMM enrolled in MAIA, regardless of frailty status.

## Supplementary information

Supplementary Information

## Data Availability

The data sharing policy of Janssen Pharmaceutical Companies of Johnson & Johnson is available at https://www.janssen.com/clinical-trials/transparency. As noted on this site, requests for access to the study data can be submitted through Yale Open Data Access (YODA) Project site at http://yoda.yale.edu.
