# Supplementary material for: Daratumumab plus lenalidomide and dexamethasone in transplant-ineligible newly diagnosed multiple myeloma: frailty subgroup analysis of MAIA
Source: Leukemia. 2022 Jan 2;36(4):1066–77. doi: 10.1038/s41375-021-01488-8 (PMC8979809; doi:10.1038/s41375-021-01488-8)
Supplement: Supplementary file 1 — Supplementary Information [file 41375_2021_1488_MOESM1_ESM.pdf]

**Supplementary Information for Facon et al. Daratumumab Plus Lenalidomide and Dexamethasone in Transplant-ineligible Newly Diagnosed Multiple Myeloma: Frailty Subgroup Analysis of MAIA.**

*Statistical analyses*

A log-rank test compared progression-free survival between treatment cohorts, and a Cox proportional hazards model was used to estimate hazard ratios and 95% confidence intervals, with treatment as the sole explanatory variable for the frailty subgroups. The Kaplan-Meier method was used to summarize time-to-event variables. A Cochran-Mantel-Haenszel chi-square test measured treatment differences in overall response rate, very good partial response or better rate, complete response or better rate, and stringent complete response rate. A Fisher's exact test was used to test treatment differences in minimal residual disease–negativity rate.

**Supplementary Table 1. Frailty assessment based on the FIRST study[a] [1]**

| Category | Score |
|---|---|
| Age | |
| ≤75 years | 0 |
| 76–80 years | 1 |
| >80 years | 2 |
| CCI[b] | |
| ≤1 | 0 |
| >1 | 1 |
| ECOG PS score | |
| 0 | 0 |
| 1 | 1 |
| ≥2 | 2 |
| Sum of scores | |
| Fit | 0 |
| Intermediate | 1 |
| Total–non-frail | 0–1 |
| Frail | ≥2 |

CCI, Charlson comorbidity index; ECOG PS, Eastern Cooperative Oncology Group performance status.

[a]This table was adapted from Table 1 from Facon T *et al*. A simplified frailty scale predicts outcomes in transplant-ineligible patients with newly diagnosed multiple myeloma treated in the FIRST (MM-020) trial. *Leukemia*. 2020;34(1):224-233. https://doi.org/10.1038/s41375-019-0539-0, which is licensed under the Creative Commons Attribution 4.0 International License (http://creativecommons.org/licenses/by/4.0).

[b]The CCI was calculated based on the definition provided by Palumbo A *et al* [2] with slight modifications. One point each was assigned for myocardial infarction (history, not electrocardiography changes only), congestive heart failure, peripheral disease (includes aortic aneurysm ≥6 cm), cerebrovascular disease (cerebrovascular accident with mild or no residua or transient ischemic attack), dementia, chronic pulmonary disease, connective tissue disease, peptic ulcer disease, mild liver disease (without portal hypertension, includes chronic hepatitis), and diabetes without end-organ damage (excludes diet-controlled alone); 2 points each were assigned for hemiplegia, moderate or severe renal disease (excludes renal disease due to multiple myeloma that resolved after the start of study treatment), diabetes with end-organ damage (retinopathy, neuropathy, nephropathy, or brittle diabetes), tumor without metastasis (exclude if >5 years from diagnosis and excludes benign tumor, basal cell carcinoma, squamous cell carcinoma of the skin, or carcinoma in situ), leukemia (acute or chronic), and lymphoma; 3 points were assigned for moderate or severe liver disease; and 6 points each were assigned for metastatic solid tumor and acquired immunodeficiency syndrome (not just human immunodeficiency virus positive).

**Supplementary Table 2. Grade 3/4 TEAEs and TEAEs with outcome of death (>1 patient; safety population)[a]**

| | Non-frail[b] | | | | | | Frail | |
|---|---|---|---|---|---|---|---|---|
| | Fit (19.9%[c]; n=145/729) | | Intermediate (34.3%[c]; n=250/729) | | Total–non-frail[b] (54.2%[c]; n=395/729) | | Frail (45.8%[c]; n=334/729) | |
| | D-Rd (18.7%[d]; n=68/364) | Rd (21.1%[e]; n=77/365) | D-Rd (35.2%[d]; n=128/364) | Rd (33.4%[e]; n=122/365) | D-Rd (53.8%[d]; n=196/364) | Rd (54.5%[e]; n=199/365) | D-Rd (46.2%[d]; n=168/364) | Rd (45.5%[e]; n=166/365) |
| Total number of patients with grade 3/4 TEAE, n (%) | 58 (85.3) | 61 (79.2) | 117 (91.4) | 104 (85.2) | 175 (89.3) | 165 (82.9) | 159 (94.6) | 148 (89.2) |
| Hematologic, n (%) | | | | | | | | |
| Neutropenia | 30 (44.1) | 22 (28.6) | 59 (46.1) | 52 (42.6) | 89 (45.4) | 74 (37.2) | 97 (57.7) | 55 (33.1) |
| Lymphopenia | 7 (10.3) | 7 (9.1) | 18 (14.1) | 14 (11.5) | 25 (12.8) | 21 (10.6) | 31 (18.5) | 18 (10.8) |
| Leukopenia | 7 (10.3) | 2 (2.6) | 11 (8.6) | 10 (8.2) | 18 (9.2) | 12 (6.0) | 22 (13.1) | 9 (5.4) |
| Anemia | 4 (5.9) | 11 (14.3) | 17 (13.3) | 24 (19.7) | 21 (10.7) | 35 (17.6) | 28 (16.7) | 40 (24.1) |
| Thrombocytopenia | 4 (5.9) | 3 (3.9) | 8 (6.3) | 12 (9.8) | 12 (6.1) | 15 (7.5) | 17 (10.1) | 18 (10.8) |
| Febrile neutropenia | 1 (1.5) | 1 (1.3) | 3 (2.3) | 6 (4.9) | 4 (2.0) | 7 (3.5) | 9 (5.4) | 4 (2.4) |
| Non-hematologic, n (%) | | | | | | | | |
| Infections | 16 (23.5) | 22 (28.6) | 46 (35.9) | 30 (24.6) | 62 (31.6) | 52 (26.1) | 70 (41.7) | 46 (27.7) |
| Pneumonia | 7 (10.3) | 5 (6.5) | 13 (10.2) | 11 (9.0) | 20 (10.2) | 16 (8.0) | 33 (19.6) | 17 (10.2) |
| Influenza | 2 (2.9) | 2 (2.6) | 5 (3.9) | 2 (1.6) | 7 (3.6) | 4 (2.0) | 5 (3.0) | 4 (2.4) |
| Lower respiratory tract infection | 2 (2.9) | 4 (5.2) | 3 (2.3) | 5 (4.1) | 5 (2.6) | 9 (4.5) | 4 (2.4) | 2 (1.2) |
| Bronchitis | 1 (1.5) | 0 | 5 (3.9) | 2 (1.6) | 6 (3.1) | 2 (1.0) | 5 (3.0) | 3 (1.8) |
| Urinary tract infection | 1 (1.5) | 2 (2.6) | 4 (3.1) | 2 (1.6) | 5 (2.6) | 4 (2.0) | 6 (3.6) | 5 (3.0) |
| Sepsis | 1 (1.5) | 5 (6.5) | 2 (1.6) | 1 (0.8) | 3 (1.5) | 6 (3.0) | 8 (4.8) | 4 (2.4) |
| Diverticulitis | 1 (1.5) | 0 | 2 (1.6) | 1 (0.8) | 3 (1.5) | 1 (0.5) | 3 (1.8) | 3 (1.8) |
| Septic shock | 1 (1.5) | 1 (1.3) | 2 (1.6) | 1 (0.8) | 3 (1.5) | 2 (1.0) | 2 (1.2) | 1 (0.6) |

| | | | | | | | | |
|---|---|---|---|---|---|---|---|---|
| Lung infection | 1 (1.5) | 0 | 1 (0.8) | 0 | 2 (1.0) | 0 | 4 (2.4) | 1 (0.6) |
| Respiratory tract infection | 1 (1.5) | 0 | 1 (0.8) | 0 | 2 (1.0) | 0 | 0 | 0 |
| Respiratory syncytial virus infection | 1 (1.5) | 1 (1.3) | 0 | 1 (0.8) | 1 (0.5) | 2 (1.0) | 0 | 0 |
| Upper respiratory tract infection | 0 | 3 (3.9) | 4 (3.1) | 0 | 4 (2.0) | 3 (1.5) | 2 (1.2) | 1 (0.6) |
| Clostridium difficile colitis | 0 | 2 (2.6) | 2 (1.6) | 1 (0.8) | 2 (1.0) | 3 (1.5) | 2 (1.2) | 0 |
| Device related infection | 0 | 0 | 2 (1.6) | 0 | 2 (1.0) | 0 | 2 (1.2) | 0 |
| Infection | 0 | 0 | 1 (0.8) | 0 | 1 (0.5) | 0 | 1 (0.6) | 2 (1.2) |
| Peritonitis | 0 | 0 | 1 (0.8) | 0 | 1 (0.5) | 0 | 0 | 2 (1.2) |
| Cellulitis | 0 | 4 (5.2) | 0 | 0 | 0 | 4 (2.0) | 3 (1.8) | 1 (0.6) |
| Erysipelas | 0 | 0 | 0 | 2 (1.6) | 0 | 2 (1.0) | 3 (1.8) | 2 (1.2) |
| Bacteremia | 0 | 0 | 0 | 1 (0.8) | 0 | 1 (0.5) | 2 (1.2) | 0 |
| Tooth abscess | 0 | 0 | 0 | 1 (0.8) | 0 | 1 (0.5) | 2 (1.2) | 0 |
| Urosepsis | 0 | 0 | 0 | 1 (0.8) | 0 | 1 (0.5) | 2 (1.2) | 0 |
| Staphylococcal bacteremia | 0 | 0 | 0 | 0 | 0 | 0 | 2 (1.2) | 1 (0.6) |
| Bacterial lower respiratory tract infection | 0 | 1 (1.3) | 0 | 1 (0.8) | 0 | 2 (1.0) | 0 | 0 |
| Cataract | 10 (14.7) | 8 (10.4) | 11 (8.6) | 9 (7.4) | 21 (10.7) | 17 (8.5) | 13 (7.7) | 19 (11.4) |
| Pulmonary embolism | 8 (11.8) | 5 (6.5) | 6 (4.7) | 9 (7.4) | 14 (7.1) | 14 (7.0) | 7 (4.2) | 5 (3.0) |
| Hypokalemia | 7 (10.3) | 5 (6.5) | 12 (9.4) | 10 (8.2) | 19 (9.7) | 15 (7.5) | 18 (10.7) | 20 (12.0) |
| Diarrhea | 5 (7.4) | 6 (7.8) | 9 (7.0) | 7 (5.7) | 14 (7.1) | 13 (6.5) | 11 (6.5) | 6 (3.6) |
| Fatigue | 4 (5.9) | 2 (2.6) | 12 (9.4) | 11 (9.0) | 16 (8.2) | 13 (6.5) | 15 (8.9) | 2 (1.2) |
| Hypertension | 4 (5.9) | 0 | 10 (7.8) | 0 | 14 (7.1) | 0 | 16 (9.5) | 13 (7.8) |
| Acute kidney injury | 4 (5.9) | 2 (2.6) | 2 (1.6) | 1 (0.8) | 6 (3.1) | 3 (1.5) | 11 (6.5) | 9 (5.4) |
| Hepatocellular injury | 3 (4.4) | 0 | 0 | 0 | 3 (1.5) | 0 | 0 | 2 (1.2) |

| | | | | | | | | |
|---|---|---|---|---|---|---|---|---|
| Hyperglycemia | 2 (2.9) | 2 (2.6) | 13 (10.2) | 4 (3.3) | 15 (7.7) | 6 (3.0) | 12 (7.1) | 8 (4.8) |
| Hypophosphatemia | 2 (2.9) | 1 (1.3) | 4 (3.1) | 1 (0.8) | 6 (3.1) | 2 (1.0) | 5 (3.0) | 1 (0.6) |
| Musculoskeletal chest pain | 2 (2.9) | 0 | 1 (0.8) | 2 (1.6) | 3 (1.5) | 2 (1.0) | 1 (0.6) | 1 (0.6) |
| Osteoarthritis | 2 (2.9) | 0 | 1 (0.8) | 2 (1.6) | 3 (1.5) | 2 (1.0) | 0 | 3 (1.8) |
| Renal failure | 2 (2.9) | 1 (1.3) | 0 | 0 | 2 (1.0) | 1 (0.5) | 4 (2.4) | 5 (3.0) |
| Myocardial ischemia | 2 (2.9) | 0 | 0 | 0 | 2 (1.0) | 0 | 0 | 0 |
| Asthenia | 1 (1.5) | 3 (3.9) | 5 (3.9) | 2 (1.6) | 6 (3.1) | 5 (2.5) | 12 (7.1) | 10 (6.0) |
| Back pain | 1 (1.5) | 1 (1.3) | 4 (3.1) | 6 (4.9) | 5 (2.6) | 7 (3.5) | 6 (3.6) | 6 (3.6) |
| Pyrexia | 1 (1.5) | 3 (3.9) | 4 (3.1) | 4 (3.3) | 5 (2.6) | 7 (3.5) | 5 (3.0) | 2 (1.2) |
| Insomnia | 1 (1.5) | 1 (1.3) | 4 (3.1) | 6 (4.9) | 5 (2.6) | 7 (3.5) | 5 (3.0) | 5 (3.0) |
| Atrial fibrillation | 1 (1.5) | 2 (2.6) | 3 (2.3) | 3 (2.5) | 4 (2.0) | 5 (2.5) | 6 (3.6) | 7 (4.2) |
| Syncope | 1 (1.5) | 0 | 3 (2.3) | 2 (1.6) | 4 (2.0) | 2 (1.0) | 4 (2.4) | 6 (3.6) |
| Generalized rash | 1 (1.5) | 1 (1.3) | 3 (2.3) | 5 (4.1) | 4 (2.0) | 6 (3.0) | 2 (1.2) | 5 (3.0) |
| Acute cholecystitis | 1 (1.5) | 0 | 3 (2.3) | 0 | 4 (2.0) | 0 | 0 | 0 |
| Hyponatremia | 1 (1.5) | 4 (5.2) | 2 (1.6) | 2 (1.6) | 3 (1.5) | 6 (3.0) | 6 (3.6) | 5 (3.0) |
| Abdominal pain | 1 (1.5) | 0 | 2 (1.6) | 0 | 3 (1.5) | 0 | 2 (1.2) | 1 (0.6) |
| Peripheral sensory neuropathy | 1 (1.5) | 0 | 2 (1.6) | 0 | 3 (1.5) | 0 | 2 (1.2) | 0 |
| Increased gamma-glutamyltransferase | 1 (1.5) | 0 | 2 (1.6) | 0 | 3 (1.5) | 0 | 1 (0.6) | 1 (0.6) |
| Diverticular perforation | 1 (1.5) | 0 | 2 (1.6) | 0 | 3 (1.5) | 0 | 0 | 2 (1.2) |
| Deep vein thrombosis | 1 (1.5) | 0 | 1 (0.8) | 5 (4.1) | 2 (1.0) | 5 (2.5) | 5 (3.0) | 3 (1.8) |
| Hyperbilirubinemia | 1 (1.5) | 0 | 1 (0.8) | 0 | 2 (1.0) | 0 | 2 (1.2) | 1 (0.6) |
| Congestive cardiac failure | 1 (1.5) | 1 (1.3) | 1 (0.8) | 1 (0.8) | 2 (1.0) | 2 (1.0) | 1 (0.6) | 4 (2.4) |
| Asthma | 1 (1.5) | 0 | 1 (0.8) | 0 | 2 (1.0) | 0 | 1 (0.6) | 0 |
| Left ventricular dysfunction | 1 (1.5) | 0 | 1 (0.8) | 0 | 2 (1.0) | 0 | 0 | 1 (0.6) |
| Hypomagnesemia | 1 (1.5) | 1 (1.3) | 0 | 1 (0.8) | 1 (0.5) | 2 (1.0) | 4 (2.4) | 0 |
| Arthralgia | 1 (1.5) | 1 (1.3) | 0 | 4 (3.3) | 1 (0.5) | 5 (2.5) | 2 (1.2) | 2 (1.2) |
| Dizziness | 1 (1.5) | 0 | 0 | 0 | 1 (0.5) | 0 | 2 (1.2) | 1 (0.6) |

| | | | | | | | | |
|---|---|---|---|---|---|---|---|---|
| Hip fracture | 1 (1.5) | 0 | 0 | 0 | 1 (0.5) | 0 | 2 (1.2) | 1 (0.6) |
| Myalgia | 1 (1.5) | 0 | 0 | 0 | 1 (0.5) | 0 | 2 (1.2) | 0 |
| Spinal pain | 1 (1.5) | 2 (2.6) | 0 | 1 (0.8) | 1 (0.5) | 3 (1.5) | 1 (0.6) | 2 (1.2) |
| Maculopapular rash | 1 (1.5) | 2 (2.6) | 0 | 2 (1.6) | 1 (0.5) | 4 (2.0) | 0 | 0 |
| Osteonecrosis of jaw | 1 (1.5) | 1 (1.3) | 0 | 2 (1.6) | 1 (0.5) | 3 (1.5) | 0 | 1 (0.6) |
| Chronic obstructive pulmonary disease | 1 (1.5) | 1 (1.3) | 0 | 0 | 1 (0.5) | 1 (0.5) | 0 | 2 (1.2) |
| Dyspnea | 0 | 0 | 5 (3.9) | 3 (2.5) | 5 (2.6) | 3 (1.5) | 7 (4.2) | 1 (0.6) |
| Spinal compression fracture | 0 | 1 (1.3) | 4 (3.1) | 0 | 4 (2.0) | 1 (0.5) | 1 (0.6) | 2 (1.2) |
| Chronic kidney disease | 0 | 1 (1.3) | 3 (2.3) | 3 (2.5) | 3 (1.5) | 4 (2.0) | 12 (7.1) | 6 (3.6) |
| Decreased weight | 0 | 2 (2.6) | 3 (2.3) | 3 (2.5) | 3 (1.5) | 5 (2.5) | 7 (4.2) | 4 (2.4) |
| Dehydration | 0 | 0 | 3 (2.3) | 2 (1.6) | 3 (1.5) | 2 (1.0) | 5 (3.0) | 1 (0.6) |
| Peripheral edema | 0 | 0 | 3 (2.3) | 0 | 3 (1.5) | 0 | 4 (2.4) | 2 (1.2) |
| Muscular weakness | 0 | 1 (1.3) | 3 (2.3) | 1 (0.8) | 3 (1.5) | 2 (1.0) | 3 (1.8) | 2 (1.2) |
| Increased alanine aminotransferase | 0 | 0 | 3 (2.3) | 1 (0.8) | 3 (1.5) | 1 (0.5) | 3 (1.8) | 3 (1.8) |
| Constipation | 0 | 0 | 3 (2.3) | 1 (0.8) | 3 (1.5) | 1 (0.5) | 3 (1.8) | 0 |
| Inguinal hernia | 0 | 1 (1.3) | 3 (2.3) | 1 (0.8) | 3 (1.5) | 2 (1.0) | 2 (1.2) | 0 |
| Acute pulmonary edema | 0 | 0 | 3 (2.3) | 0 | 3 (1.5) | 0 | 2 (1.2) | 2 (1.2) |
| Pain in extremity | 0 | 0 | 3 (2.3) | 0 | 3 (1.5) | 0 | 2 (1.2) | 0 |
| Hypocalcemia | 0 | 2 (2.6) | 2 (1.6) | 3 (2.5) | 2 (1.0) | 5 (2.5) | 4 (2.4) | 3 (1.8) |
| Bone pain | 0 | 1 (1.3) | 2 (1.6) | 2 (1.6) | 2 (1.0) | 3 (1.5) | 3 (1.8) | 4 (2.4) |
| Cognitive disorder | 0 | 0 | 2 (1.6) | 1 (0.8) | 2 (1.0) | 1 (0.5) | 3 (1.8) | 0 |
| Atrial flutter | 0 | 0 | 2 (1.6) | 1 (0.8) | 2 (1.0) | 1 (0.5) | 2 (1.2) | 1 (0.6) |
| Femoral neck fracture | 0 | 0 | 2 (1.6) | 1 (0.8) | 2 (1.0) | 1 (0.5) | 2 (1.2) | 0 |
| Rash | 0 | 0 | 2 (1.6) | 1 (0.8) | 2 (1.0) | 1 (0.5) | 2 (1.2) | 0 |
| Squamous cell carcinoma of the skin | 0 | 0 | 2 (1.6) | 0 | 2 (1.0) | 0 | 2 (1.2) | 2 (1.2) |
| Hypogammaglobulinemia | 0 | 0 | 2 (1.6) | 0 | 2 (1.0) | 0 | 2 (1.2) | 0 |

| | | | | | | | | |
|---|---|---|---|---|---|---|---|---|
| Femur fracture | 0 | 1 (1.3) | 2 (1.6) | 1 (0.8) | 2 (1.0) | 2 (1.0) | 1 (0.6) | 3 (1.8) |
| Decreased appetite | 0 | 0 | 2 (1.6) | 1 (0.8) | 2 (1.0) | 1 (0.5) | 1 (0.6) | 1 (0.6) |
| Increase aspartate aminotransferase | 0 | 0 | 2 (1.6) | 0 | 2 (1.0) | 0 | 1 (0.6) | 2 (1.2) |
| Basal cell carcinoma | 0 | 0 | 2 (1.6) | 0 | 2 (1.0) | 0 | 1 (0.6) | 1 (0.6) |
| Gastrointestinal hemorrhage | 0 | 2 (2.6) | 2 (1.6) | 0 | 2 (1.0) | 2 (1.0) | 0 | 2 (1.2) |
| Cholelithiasis | 0 | 0 | 2 (1.6) | 0 | 2 (1.0) | 0 | 0 | 0 |
| Agitation | 0 | 0 | 2 (1.6) | 0 | 2 (1.0) | 0 | 0 | 0 |
| Confusional state | 0 | 0 | 1 (0.8) | 0 | 1 (0.5) | 0 | 6 (3.6) | 3 (1.8) |
| Cardiac failure | 0 | 0 | 1 (0.8) | 2 (1.6) | 1 (0.5) | 2 (1.0) | 5 (3.0) | 4 (2.4) |
| Hypoxia | 0 | 0 | 1 (0.8) | 0 | 1 (0.5) | 0 | 4 (2.4) | 2 (1.2) |
| Renal impairment | 0 | 0 | 1 (0.8) | 1 (0.8) | 1 (0.5) | 1 (0.5) | 3 (1.8) | 8 (4.8) |
| Cerebrovascular accident | 0 | 0 | 1 (0.8) | 1 (0.8) | 1 (0.5) | 1 (0.5) | 3 (1.8) | 4 (2.4) |
| Hypotension | 0 | 1 (1.3) | 1 (0.8) | 1 (0.8) | 1 (0.5) | 2 (1.0) | 2 (1.2) | 2 (1.2) |
| Increased c-reactive protein | 0 | 1 (1.3) | 1 (0.8) | 0 | 1 (0.5) | 1 (0.5) | 2 (1.2) | 0 |
| Seizure | 0 | 0 | 1 (0.8) | 0 | 1 (0.5) | 0 | 2 (1.2) | 2 (1.2) |
| Ischemic colitis | 0 | 0 | 1 (0.8) | 0 | 1 (0.5) | 0 | 2 (1.2) | 0 |
| Thoracic vertebral fracture | 0 | 0 | 1 (0.8) | 0 | 1 (0.5) | 0 | 2 (1.2) | 0 |
| Hypoalbuminemia | 0 | 1 (1.3) | 1 (0.8) | 2 (1.6) | 1 (0.5) | 3 (1.5) | 1 (0.6) | 1 (0.6) |
| Altered mood | 0 | 1 (1.3) | 1 (0.8) | 1 (0.8) | 1 (0.5) | 2 (1.0) | 1 (0.6) | 2 (1.2) |
| Ischemic stroke | 0 | 1 (1.3) | 1 (0.8) | 1 (0.8) | 1 (0.5) | 2 (1.0) | 1 (0.6) | 1 (0.6) |
| Depression | 0 | 0 | 1 (0.8) | 2 (1.6) | 1 (0.5) | 2 (1.0) | 1 (0.6) | 2 (1.2) |
| Sciatica | 0 | 0 | 1 (0.8) | 2 (1.6) | 1 (0.5) | 2 (1.0) | 1 (0.6) | 0 |
| Hypercalcemia | 0 | 0 | 1 (0.8) | 1 (0.8) | 1 (0.5) | 1 (0.5) | 0 | 2 (1.2) |
| Nausea | 0 | 0 | 0 | 1 (0.8) | 0 | 1 (0.5) | 5 (3.0) | 1 (0.6) |
| Non-cardiac chest pain | 0 | 2 (2.6) | 0 | 2 (1.6) | 0 | 4 (2.0) | 4 (2.4) | 1 (0.6) |
| Fall | 0 | 0 | 0 | 0 | 0 | 0 | 4 (2.4) | 2 (1.2) |
| Hyperuricemia | 0 | 0 | 0 | 2 (1.6) | 0 | 2 (1.0) | 3 (1.8) | 3 (1.8) |

| | | | | | | | | |
|---|---|---|---|---|---|---|---|---|
| Acute pancreatitis | 0 | 0 | 0 | 0 | 0 | 0 | 3 (1.8) | 1 (0.6) |
| Muscle spasms | 0 | 2 (2.6) | 0 | 1 (0.8) | 0 | 3 (1.5) | 2 (1.2) | 1 (0.6) |
| Anxiety | 0 | 1 (1.3) | 0 | 3 (2.5) | 0 | 4 (2.0) | 2 (1.2) | 0 |
| Vomiting | 0 | 0 | 0 | 2 (1.6) | 0 | 2 (1.0) | 2 (1.2) | 0 |
| Musculoskeletal pain | 0 | 0 | 0 | 1 (0.8) | 0 | 1 (0.5) | 2 (1.2) | 0 |
| Bradycardia | 0 | 0 | 0 | 0 | 0 | 0 | 2 (1.2) | 2 (1.2) |
| Ventricular tachycardia | 0 | 0 | 0 | 0 | 0 | 0 | 2 (1.2) | 1 (0.6) |
| Upper abdominal pain | 0 | 0 | 0 | 0 | 0 | 0 | 2 (1.2) | 0 |
| Venous thrombosis | 0 | 0 | 0 | 0 | 0 | 0 | 2 (1.2) | 0 |
| Groin pain | 0 | 0 | 0 | 0 | 0 | 0 | 2 (1.2) | 0 |
| Bronchospasm | 0 | 0 | 0 | 0 | 0 | 0 | 2 (1.2) | 0 |
| Adenocarcinoma of colon | 0 | 0 | 0 | 0 | 0 | 0 | 2 (1.2) | 0 |
| Hyperkalemia | 0 | 1 (1.3) | 0 | 3 (2.5) | 0 | 4 (2.0) | 1 (0.6) | 3 (1.8) |
| Coronary artery disease | 0 | 1 (1.3) | 0 | 1 (0.8) | 0 | 2 (1.0) | 1 (0.6) | 0 |
| General physical health deterioration | 0 | 0 | 0 | 3 (2.5) | 0 | 3 (1.5) | 1 (0.6) | 5 (3.0) |
| Colitis | 0 | 0 | 0 | 0 | 0 | 0 | 1 (0.6) | 5 (3.0) |
| Urinary retention | 0 | 0 | 0 | 0 | 0 | 0 | 1 (0.6) | 3 (1.8) |
| Rectal hemorrhage | 0 | 0 | 0 | 0 | 0 | 0 | 1 (0.6) | 2 (1.2) |
| Rib fracture | 0 | 0 | 0 | 0 | 0 | 0 | 1 (0.6) | 2 (1.2) |
| Esophagitis | 0 | 2 (2.6) | 0 | 0 | 0 | 2 (1.0) | 0 | 1 (0.6) |
| Drug reaction with eosinophilia and systemic symptoms | 0 | 1 (1.3) | 0 | 1 (0.8) | 0 | 2 (1.0) | 0 | 2 (1.2) |
| Pathological fracture | 0 | 1 (1.3) | 0 | 1 (0.8) | 0 | 2 (1.0) | 0 | 0 |
| Vertigo | 0 | 1 (1.3) | 0 | 1 (0.8) | 0 | 2 (1.0) | 0 | 0 |
| Acute coronary syndrome | 0 | 0 | 0 | 2 (1.6) | 0 | 2 (1.0) | 0 | 2 (1.2) |
| Bone lesion | 0 | 0 | 0 | 2 (1.6) | 0 | 2 (1.0) | 0 | 0 |
| Fecaloma | 0 | 0 | 0 | 0 | 0 | 0 | 0 | 2 (1.2) |

| | | | | | | | | |
|---|---|---|---|---|---|---|---|---|
| Gastric ulcer | 0 | 0 | 0 | 0 | 0 | 0 | 0 | 2 (1.2) |
| Large intestinal ulcer | 0 | 0 | 0 | 0 | 0 | 0 | 0 | 2 (1.2) |
| Memory impairment | 0 | 0 | 0 | 0 | 0 | 0 | 0 | 2 (1.2) |
| Irritability | 0 | 0 | 0 | 0 | 0 | 0 | 0 | 2 (1.2) |
| Skin ulcer | 0 | 0 | 0 | 0 | 0 | 0 | 0 | 2 (1.2) |
| Cholestasis | 0 | 0 | 0 | 0 | 0 | 0 | 0 | 2 (1.2) |
| Total number of patients with a TEAE with outcome of death, n (%) | 1 (1.5) | 3 (3.9) | 6 (4.7) | 4 (3.3) | 7 (3.6) | 7 (3.5) | 20 (11.9) | 20 (12.0) |
| General physical health deterioration | 0 | 0 | 0 | 1 (0.8) | 0 | 1 (0.5) | 2 (1.2) | 1 (0.6) |
| Pneumonia | 0 | 0 | 0 | 0 | 0 | 0 | 2 (1.2) | 3 (1.8) |
| Myocardial infarction | 0 | 0 | 0 | 2 (1.6) | 0 | 2 (1.0) | 1 (0.6) | 1 (0.6) |
| Cardiac arrest | 0 | 0 | 0 | 0 | 0 | 0 | 1 (0.6) | 2 (1.2) |
| Sepsis | 0 | 0 | 0 | 0 | 0 | 0 | 0 | 3 (1.8) |

TEAE, treatment emergent adverse event; D-Rd, daratumumab plus lenalidomide/dexamethasone; Rd, lenalidomide/dexamethasone.

[a]Percentages in the table were calculated using the number of patients in each treatment cohort per frailty subgroup of the safety population (fit: D-Rd, n=68; Rd, n=77; intermediate: D-Rd, n=128; Rd, n=122; total–non-frail: D-Rd, n=196; Rd, n=199; frail: D-Rd; n=168; Rd, n=166) as the denominator, unless otherwise indicated.

[b]Non-frail subgroup consists of fit and intermediate patients.

[c]Percentage was calculated using the number of patients in the safety population as the denominator.

[d]Percentage was calculated using the number of patients in the D-Rd cohort of the safety population as the denominator.

[e]Percentage was calculated using the number of patients in the Rd cohort of the safety population as the denominator.

**Supplementary Table 3. Summary of growth factor use (ITT population)[a]**

| | Non-frail[b] | | | | | | Frail | |
|---|---|---|---|---|---|---|---|---|
| | Fit (19.8%[c]; n=146/737) | | Intermediate (33.9%[c]; n=250/737) | | Total–non-frail[b] (53.7%[c]; n=396/737) | | Frail (46.3%[c]; n=341/737) | |
| | D-Rd (18.5%[d]; n=68/368) | Rd (21.1%[e]; n=78/369) | D-Rd (34.8%[d]; n=128/368) | Rd (33.1%[e]; n=122/369) | D-Rd (53.3%[d]; n=196/368) | Rd (54.2%[e]; n=200/369) | D-Rd (46.7%[d]; n=172/368) | Rd (45.8%[e]; n=169/369) |
| Total number of patients who received growth factors, n (%) | 23 (33.8) | 14 (17.9) | 38 (29.7) | 30 (24.6) | 61 (31.1) | 44 (22.0) | 66 (38.4) | 40 (23.7) |
| Growth factor, n (%) | | | | | | | | |
| Filgrastim | 18 (26.5) | 7 (9.0) | 26 (20.3) | 24 (19.7) | 44 (22.4) | 31 (15.5) | 49 (28.5) | 31 (18.3) |
| Lenograstim | 7 (10.3) | 3 (3.8) | 7 (5.5) | 5 (4.1) | 14 (7.1) | 8 (4.0) | 15 (8.7) | 5 (3.0) |
| Granulocyte colony-stimulating factor | 4 (5.9) | 3 (3.8) | 7 (5.5) | 4 (3.3) | 11 (5.6) | 7 (3.5) | 7 (4.1) | 7 (4.1) |
| Pegfilgrastim | 1 (1.5) | 2 (2.6) | 2 (1.6) | 3 (2.5) | 3 (1.5) | 5 (2.5) | 4 (2.3) | 3 (1.8) |
| Granulocyte macrophage colony-stimulating factor | 1 (1.5) | 1 (1.3) | 2 (1.6) | 0 | 3 (1.5) | 1 (0.5) | 1 (0.6) | 0 |

D-Rd, daratumumab plus lenalidomide/dexamethasone; Rd, lenalidomide/dexamethasone.

[a] Percentages in the table were calculated using the number of patients in each treatment cohort per frailty subgroup of the ITT population (fit: D-Rd, n=68; Rd, n=78; intermediate: D-Rd, n=128; Rd, n=122; total–non-frail: D-Rd, n=196; Rd, n=200; frail: D-Rd; n=172; Rd, n=169) as the denominator, unless otherwise indicated.

[b]Non-frail subgroup consists of fit and intermediate patients.

[c]Percentage was calculated using the number of patients in the ITT population as the denominator.

[d]Percentage was calculated using the number of patients in the D-Rd cohort of the ITT population as the denominator.

[e]Percentage was calculated using the number of patients in the Rd cohort of the ITT population as the denominator.

**Supplementary Figure 1. PFS by lenalidomide starting dose[a] in the (A) ITT population, (B) total–non-frail subgroup, and (C) frail subgroup.**

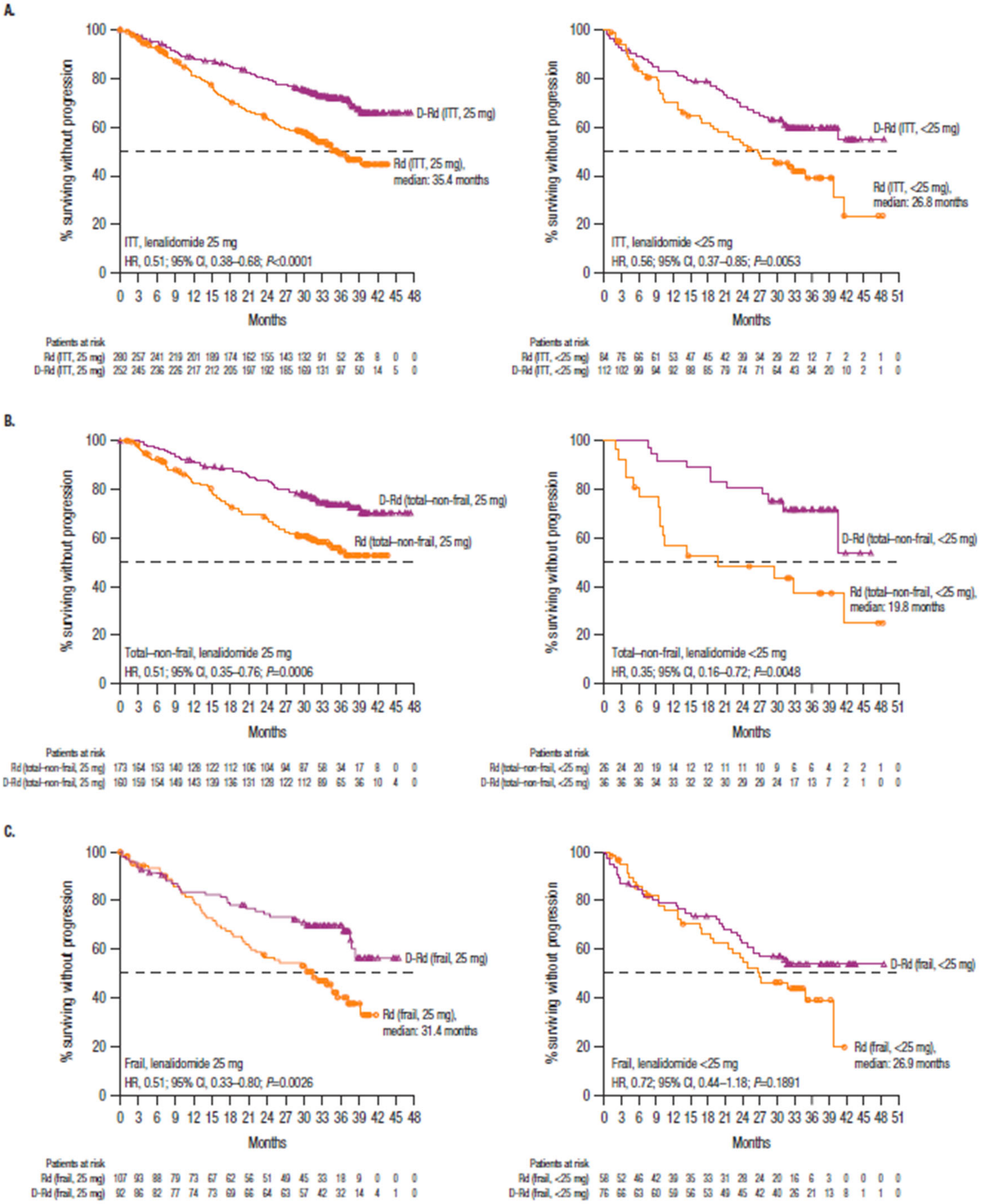

PFS, progression-free survival; ITT, intent-to-treat; D-Rd, daratumumab plus lenalidomide/dexamethasone; Rd, lenalidomide/dexamethasone; HR, hazard ratio; CI, confidence interval; CrCl, creatinine clearance.
[a]In most cases, patients received a reduced starting dose of lenalidomide (<25 mg) due to a CrCl of 30 to 50 mL/min per the study protocol, which recommended a lenalidomide dose of 10 mg if CrCl was 30 to 50 mL/min.

**References**


1. Facon T, Dimopoulos MA, Meuleman N, Belch A, Mohty M, Chen WM *et al*. A simplified frailty scale predicts outcomes in transplant-ineligible patients with newly diagnosed multiple myeloma treated in the FIRST (MM-020) trial. *Leukemia* 2020; **34**: 224–233.
2. Palumbo A, Bringhen S, Mateos MV, Larocca A, Facon T, Kumar SK *et al*. Geriatric assessment predicts survival and toxicities in elderly myeloma patients: an International Myeloma Working Group report. *Blood* 2015; **125**: 2068–2074.
